# Spurious prospective effects between general and domain-specific self-esteem: A reanalysis of a meta-analysis of longitudinal studies

**DOI:** 10.1371/journal.pone.0298158

**Published:** 2024-02-13

**Authors:** Kimmo Sorjonen, Bo Melin

**Affiliations:** Department of Clinical Neuroscience, Karolinska Institutet, Stockholm, Sweden; Dr DY Patil Medical College, Hospital and Research Center, INDIA

## Abstract

A recent meta-analysis, of 38 studies with data from 43 independent samples (total *N* = 24,668), claimed evidence for positive reciprocal prospective effects, and hence for both top-down and bottom-up processes, between general and domain-specific self-esteem. However, the meta-analytic cross-lagged effects were estimated while adjusting for a prior measurement of the outcome variable and it is known that such adjusted cross-lagged effects may be spurious due to correlations with residuals and regression to the mean. In the present reanalyses, we found all of the prospective effects to be spurious. Consequently, claims about increasing prospective effects and top-down and bottom-up processes between general and domain-specific self-esteem can be questioned. It is important for researchers to be aware of the limitations of cross-lagged panel analyses, and of analyses of correlational data in general, in order not to overinterpret findings.

## Introduction

Researchers have suggested that low self-esteem may increase the risk for various negative outcomes, including poor physical and mental health [1,2], worse work experiences and economic prospects [2,3], low quality of social relations [4], and criminal behavior [2]. Consequently, it may be important to grasp determinants of self-esteem. Although self-esteem is often regarded as a general construct, it has been suggested that people’s self-esteem (or the related “self-concept”) can vary between more specific domains, e.g. academic ability and physical appearance [5,6]. A top-down model claims influence from general to domain-specific self-esteem [7,8] while a bottom-up model suggests effects in the opposite direction [5,9,10].

Dapp et al. [11] estimated meta-analytic prospective effects of initial general self-esteem on subsequent domain-specific self-esteem while adjusting for a prior measurement of the same domain-specific self-esteem, and vice versa. Dapp et al. did this for eight different domain-specific self-esteems, namely academic abilities, physical appearance, athletic abilities, math abilities, morality, romantic relationships, social acceptance, and verbal abilities. With a few exceptions, both the prospective adjusted effect of general self-esteem on subsequent domain-specific self-esteem and the prospective adjusted effect of domain-specific self-esteem on subsequent general self-esteem were positive and statistically significant. Dapp et al. concluded that their findings provided robust evidence for reciprocal effects, and consequently for both top-down and bottom-up processes, between general and domain-specific self-esteem.

However, it is known that cross-lagged effects while adjusting for a prior measurement of the outcome may be spurious due to correlations with residuals and regression to the mean [12–15]. As an example, let us assume that cabdrivers tend to drive more than gardeners. Consequently, if a cabdriver and a gardener has driven equally much a particular week, we may suspect that the cabdriver has driven less than usually, i.e. experienced a negative residual, or that the gardener has driven more than usually, i.e. experienced a positive residual. However, as residuals tend to regress toward a mean value of zero between measurements, we should expect a more positive change in driving to the subsequent week for the cabdriver compared with the gardener. On group level, we should expect a positive effect of a dichotomous “cabdriver vs. gardener” variable on subsequent driving while adjusting for initial driving even if no group-level change has taken place. As regression to the mean is independent of the direction of time, if the effect is spurious, we should also expect a positive effect of “cabdriver vs. gardener” on initial driving while adjusting for subsequent driving. The impact of correlations with residuals and regression to the mean on adjusted cross-lagged effects is demonstrated in a simulation included in one of our recent publications [16].

The objective of the present reanalyses was to evaluate if the meta-analytic findings by Dapp et al. [11] really provided evidence for reciprocal prospective effects, and consequently for top-down and bottom-up processes, between general and domain-specific self-esteem or if the findings may have been spurious due to correlations with residuals and regression to the mean. As mentioned above, research has indicated potential negative consequences of low self-esteem and clinical practitioners and decision-makers may think that increased self-esteem could be one way to avoid or alleviate those negative consequences. Therefore, it is important to scrutinize research in the area, and to identify effects that could be spurious, in order to help avoid investments that may not be the best use of limited resources.

## Method

We refer to Dapp et al. [11] for more comprehensive information on selection of studies, descriptive data, etc. In short, Dapp et al. extracted six zero-order correlations between general and eight types of domain-specific self-esteem measured at two occasions from 38 studies with data from 43 independent samples (total *N* = 24,668, mean age at time 1 = 12.6 years (*SD* = 3.5 years), mean proportion of female participants = 53% (*SD* = 22%)). The domain-specific self-esteems were academic abilities, physical appearance, athletic abilities, math abilities, morality, romantic relationships, social acceptance, and verbal abilities. Dapp et al. estimated meta-analytic cross-lagged effects between general and domain-specific self-esteem while adjusting for a prior measurement of the outcome with meta-analytic structural equation modeling (MASEM).

Using zero-order correlations provided by Dapp et al. (at the Open Science Framework at https://osf.io/beu29/, from where we retrieved data 2023-04-21) and Eq 1 [17], we estimated meta-analytic effects of initial global self-esteem on subsequent domain-specific self-esteem while adjusting for initial domain-specific self-esteem, and vice versa. Here, both a hypothesis of true prospective effects and a hypothesis of spurious prospective effects due to correlations with residuals and regression to the mean predicted positive effects (Table 1, rows 1 and 4).


$$E\left|\beta_{X1,Y2.Y1}\right|=\frac{r_{X1,Y2}-r_{X1,Y1}r_{Y1,Y2}}{1-r_{X1,Y1}^{2}}$$ (1)

**Table 1:** Predicted sign of effects between general and domain-specific self-esteem according to a hypothesis of true increasing reciprocal effects and a hypothesis of spuriousness.

Effect^1^	True	Spurious
1. β(g1,s2.s1)	Positive	Positive
2. β(g1,s1.s2)	Negative	Positive
3. β(g1,s2-s1)	Positive	Zero or Negative
4. β(s1,g2.g1)	Positive	Positive
5. β(s1,g1.g2)	Negative	Positive
6. β(s1,g2-g1)	Positive	Zero or Negative

Note: g = general self-esteem; s = domain-specific self-esteem; 1 = time 1; 2 = time 2;

^1^ the variables are given in the order predictor, outcome, and covariate.

Additionally, we used Eq 1 to estimate meta-analytic effects of initial general self-esteem on initial domain-specific self-esteem while adjusting for subsequent domain-specific self-esteem, and vice versa. Here, a hypothesis of true increasing prospective effects predicted negative effects, which would mean that among individuals with the same subsequent domain-specific self-esteem, those with high initial general self-esteem had had lower initial domain-specific self-esteem and had, consequently, experienced a larger increase in domain-specific self-esteem between measurements compared with those with the same subsequent domain-specific self-esteem but with lower initial general self-esteem. Similarly, a negative effect would mean that those with high initial domain-specific self-esteem had experienced a larger subsequent increase in general self-esteem compared with those with the same subsequent general self-esteem but with lower initial domain-specific self-esteem. Contrarily, as regression to the mean is independent of the direction of time, a hypothesis of spuriousness predicted these effects to be positive (Table 1, rows 2 and 5).

Moreover, we used Eq 2 [18] to estimate meta-analytic effects of initial general self-esteem on the subsequent domain-specific self-esteem–initial domain-specific self-esteem difference, and vice versa. Here, a hypothesis of true increasing prospective effects predicted positive effects. Contrarily, a hypothesis of spuriousness predicted these effects to be either close to zero (if concurrent, *r*_*X1*,*Y1*_, and cross-lagged, *r*_*X1*,*Y2*_, correlations were approximately equally strong) or negative (if concurrent correlations were stronger than cross-lagged correlations) (Table 1, rows 3 and 6).


$$E\left|\beta_{X1,Y2-Y1}\right|=\frac{r_{X1,Y2}-r_{X1,Y1}}{\sqrt{2\left(1-r_{Y1,Y2}\right)}}$$ (2)

We conducted a multilevel random effects meta-analysis for each of the six effects in Table 1 for each of the eight domain-specific self-esteems, i.e. 48 meta-analyses in total. Different effects from the same study were aggregated with a multilevel approach. Then, a random meta-analytic effect, with a 95% confidence interval, was estimated across the independent effect sizes. Analyses were conducted on Fisher’s z-transformed standardized regression effects, but these were inverted back to non-transformed effects for presentations. Analyses were conducted with R 4.1.3 statistical software [19] employing the metafor package [20]. Data, a list of studies included in the meta-analyses, forest-plots, and an analysis script are available at the Open Science Framework at https://osf.io/qu2hd/.

## Results

**Fig 1:**
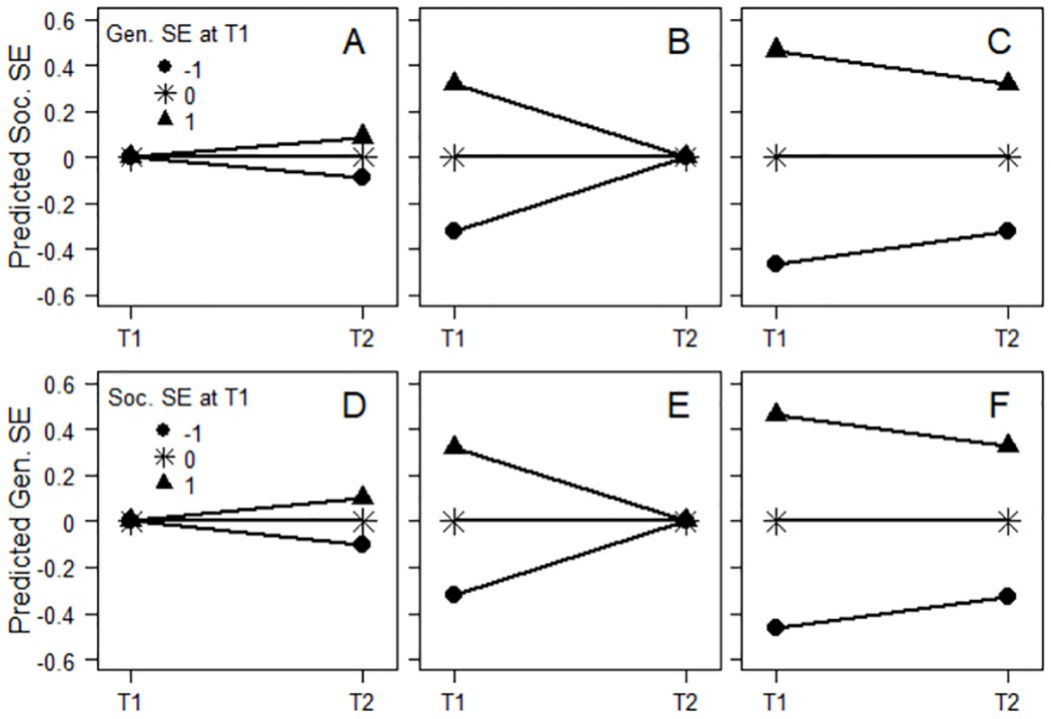
Predicted social and general self-esteem. Predicted social (A-C) and general (D-F) self-esteem at two occasions, separately for those with high (*Z* = 1), average, and low (*Z* = -1) general (A-C) and social (D-F) self-esteem at T1. Separately for situations when conditioning on average level of the outcome at T1 (A and D), average level of the outcome at T2 (B and E), and when not conditioning on the outcome (C and F).

Meta-analytic findings are presented in Table 2. Most estimated effects exhibited statistically significant, although not necessarily high, heterogeneity, as estimated by Cochran’s *Q* and *I*^*2*^, which estimates percentage of variation across effects attributable to heterogeneity rather than random variance. With two exceptions, the effects of initial general self-esteem on subsequent domain-specific self-esteem while adjusting for initial domain-specific self-esteem (β(g1,s2.s1)), and vice versa (β(s1,g2.g1)), were positive and statistically significant. This suggested a larger subsequent increase in domain-specific self-esteem, for example social acceptance, for those with high, compared with low, initial general self-esteem (Fig 1A), and vice versa (Fig 1D).

**Table 2:** Meta-analytic standardized regression effects between general and eight types of domain-specific self-esteem measured at two occasions.

Domain/Eff^1^	*K*	*NE*	*N*	β (95% CI)	*Q* (df)	*I*^*2*^ (95% CI)
Academic						
β(g1,s2.s1)	25	26	17534	0.10 (0.07; 0.14)*	92 (24)*	0 (0; 45)
β(g1,s1.s2)	25	26	17534	0.37 (0.31; 0.44)*	677 (24)*	0 (0; 48)
β(g1,s2-s1)	25	26	17534	-0.17 (-0.21; -0.13)*	147 (24)*	0 (0; 48)
β(s1,g2.g1)	25	26	17534	0.12 (0.09; 0.15)*	100 (24)*	0 (0; 49)
β(s1,g1.g2)	25	26	17534	0.40 (0.33; 0.46)*	711 (24)*	0 (0; 47)
β(s1,g2-g1)	25	26	17534	-0.17 (-0.21; -0.14)*	112 (24)*	0 (0; 39)
Appearance						
β(g1,s2.s1)	21	23	14855	0.11 (0.07; 0.14)*	57 (20)*	9 (0; 60)
β(g1,s1.s2)	21	23	14855	0.47 (0.41; 0.52)*	336 (20)*	0 (0; 50)
β(g1,s2-s1)	21	23	14855	-0.21 (-0.26; -0.17)*	98 (20)*	3 (0; 55)
β(s1,g2.g1)	22	25	15118	0.19 (0.15; 0.23)*	157 (21)*	0 (0; 44)
β(s1,g1.g2)	22	25	15118	0.49 (0.43; 0.54)*	446 (21)*	0 (0; 50)
β(s1,g2-g1)	22	25	15118	-0.18 (-0.22; -0.14)*	96 (21)*	0 (0; 46)
Athletic						
β(g1,s2.s1)	16	17	12084	0.05 (0.03; 0.06)*	15 (15)	0 (0; 78)
β(g1,s1.s2)	16	17	12084	0.18 (0.15; 0.22)*	38 (15)*	13 (0; 70)
β(g1,s2-s1)	16	17	12084	-0.08 (-0.10; -0.06)*	24 (15)	32 (0; 86)
β(s1,g2.g1)	16	17	12084	0.07 (0.03; 0.11)*	51 (15)*	0 (0; 59)
β(s1,g1.g2)	16	17	12084	0.23 (0.19; 0.27)*	42 (15)*	12 (0; 69)
β(s1,g2-g1)	16	17	12084	-0.11 (-0.15; -0.07)*	54 (15)*	5 (0; 63)
Math						
β(g1,s2.s1)	4	4	6643	0.01 (-0.02; 0.04)	4 (3)	22 (0; 98)
β(g1,s1.s2)	4	4	6643	0.27 (0.16; 0.36)*	33 (3)*	93 (72; 100)
β(g1,s2-s1)	4	4	6643	-0.18 (-0.23; -0.12)*	12 (3)*	72 (9; 98)
β(s1,g2.g1)	4	4	6643	0.10 (0.08; 0.13)*	0 (3)	0 (0; 18)
β(s1,g1.g2)	4	4	6643	0.27 (0.17; 0.37)*	27 (3)*	92 (71; 99)
β(s1,g2-g1)	4	4	6643	-0.11 (-0.17; -0.05)*	10 (3)*	75 (0; 99)
Morality						
β(g1,s2.s1)	14	15	8050	0.10 (0.06; 0.14)*	30 (13)*	0 (0; 54)
β(g1,s1.s2)	14	15	8050	0.35 (0.30; 0.41)*	153 (13)*	0 (0; 69)
β(g1,s2-s1)	14	15	8050	-0.15 (-0.21; -0.10)*	46 (13)*	2 (0; 63)
β(s1,g2.g1)	14	15	8050	0.08 (0.05; 0.11)*	19 (13)	10 (0; 69)
β(s1,g1.g2)	14	15	8050	0.34 (0.28; 0.39)*	137 (13)*	0 (0; 69)
β(s1,g2-g1)	14	15	8050	-0.16 (-0.20; -0.12)*	42 (13)*	0 (0; 58)
Romantic						
β(g1,s2.s1)	11	11	10235	0.10 (0.07; 0.13)*	14 (10)	34 (0; 77)
β(g1,s1.s2)	11	11	10235	0.20 (0.14; 0.26)*	63 (10)*	87 (70; 96)
β(g1,s2-s1)	11	11	10235	-0.06 (-0.09; -0.02)*	18 (10)	46 (0; 90)
β(s1,g2.g1)	11	11	10235	0.06 (0.02; 0.09)*	20 (10)*	48 (0; 80)
β(s1,g1.g2)	11	11	10235	0.21 (0.16; 0.26)*	37 (10)*	78 (48; 94)
β(s1,g2-g1)	11	11	10235	-0.09 (-0.12; -0.07)*	14 (10)	30 (0; 82)
Social						
β(g1,s2.s1)	27	31	19791	0.09 (0.06; 0.11)*	94 (26)*	0 (0; 49)
β(g1,s1.s2)	27	31	19791	0.32 (0.27; 0.37)*	378 (26)*	0 (0; 43)
β(g1,s2-s1)	27	31	19791	-0.14 (-0.18; -0.11)*	191 (26)*	0 (0; 45)
β(s1,g2.g1)	28	32	20174	0.10 (0.07; 0.13)*	134 (27)*	4 (0; 50)
β(s1,g1.g2)	28	32	20174	0.32 (0.27; 0.37)*	443 (27)*	0 (0; 44)
β(s1,g2-g1)	28	32	20174	-0.14 (-0.18; -0.10)*	171 (27)*	2 (0; 48)
Verbal						
β(g1,s2.s1)	4	4	6643	0.09 (-0.01; 0.19)	15 (3)*	91 (55; 100)
β(g1,s1.s2)	4	4	6643	0.24 (0.08; 0.38)*	58 (3)*	97 (89; 100)
β(g1,s2-s1)	4	4	6643	-0.08 (-0.24; 0.07)	43 (3)*	97 (88; 100)
β(s1,g2.g1)	4	4	6643	0.08 (0.05; 0.10)*	2 (3)	3 (0; 90)
β(s1,g1.g2)	4	4	6643	0.28 (0.14; 0.41)*	59 (3)*	96 (86; 100)
β(s1,g2-g1)	4	4	6643	-0.14 (-0.22; -0.07)*	19 (3)*	85 (44; 99)

Note: *K* = number of studies; *NE* = number of effects; *N* = total sample size; *Q* = Cochran’s Q; *I*^*2*^ = percentage of variation due to heterogeneity rather than randomness; g = general self-esteem; s = domain-specific self-esteem; 1 = time 1; 2 = time 2;

^1^ the variables are given in the order predictor, outcome, and covariate;

* *p* < 0.05.

However, all effects of initial general self-esteem on initial domain-specific self-esteem while adjusting for subsequent domain-specific self-esteem (β(g1,s1.s2)), and vice versa (β(s1,g1.g2)), were positive and statistically significant (Table 2). This suggested, contrarily, a larger subsequent decrease in domain-specific self-esteem for those with high, compared with low, initial general self-esteem (Fig 1B), and vice versa (Fig 1E).

Furthermore, with one exception, the effects of initial general self-esteem on the subsequent domain-specific self-esteem–initial domain-specific self-esteem difference (β(g1,s2-s1)), and vice versa (β(s1,g2-g1)), were negative and statistically significant (Table 2). This suggested, again, that those with high initial general self-esteem had experienced a larger subsequent decrease in domain-specific self-esteem compared with those with low initial general self-esteem (Fig 1C), and vice versa (Fig 1F). In summary, the findings agreed with a hypothesis of spurious prospective effects due to correlations with residuals and regression to the mean rather than with a hypothesis of true increasing prospective effects (compare effects in Table 2 with predictions in Table 1).

## Discussion

The present study set out to evaluate if the meta-analytic findings by Dapp et al. [11] really provided evidence for reciprocal prospective effects, and consequently for top-down and bottom-up processes, between general and domain-specific self-esteem. The present findings suggested that findings by Dapp et al. probably were spurious due to correlations with residuals and regression to the mean. Consequently, the conclusion by Dapp et al. can be challenged.

In the present reanalyses we found, with a few exceptions, positive meta-analytic reciprocal prospective effects between general and domain-specific self-esteem. This could indicate, as suggested by Dapp et al. [11], a reinforcing loop of self-esteem including both bottom-up and top-down processes. However, we also found positive effects of initial general self-esteem on initial domain-specific self-esteem while adjusting for subsequent domain-specific self-esteem, and vice versa. Moreover, we found negative effects of initial general self-esteem on the subsequent domain-specific self-esteem–initial domain-specific self-esteem difference, and vice versa. These latter findings indicated, contrarily, degrading influence between general and domain-specific self-esteem. These contradictory findings of simultaneous reinforcing and degrading effects suggested that the prospective effects between general and domain-specific self-esteem were spurious, probably due to correlations with residuals and regression to the mean.

As an example, picture two individuals, A and B, with the same initial social self-esteem but who differ in initial general self-esteem, with A having a higher score. Due to the positive association between general and social self-esteem (*r* = 0.45 according to the meta-analytic estimation by Dapp et al. [11]) in combination with assumed less than perfect reliability in measurements, we should assume a higher true social self-esteem and, consequently, a more negative residual in the initial measurement of social self-esteem for A compared with B. Furthermore, as residuals tend to regress toward a mean value of zero between measurements, we should expect a more positive, but spurious, subsequent change in social self-esteem for A compared with B.

This study is part of a series where we have reanalyzed meta-analyses using cross-lagged panel analyses. All claimed prospective effects in Table 3 were found to be spurious. An overall message in these challenging studies is that cross-lagged effects while adjusting for a prior measurement of the outcome often do not prove anything over and above a cross-sectional correlation combined with less than perfect reliability in measurements. And the cross-sectional correlation could be spurious due to confounding by a third variable, common method bias, etc. The limitation is not alleviated by meta-analytic aggregation of several cross-lagged effects. It is important for researchers to be aware of this limitation in order not to overinterpret findings, something that appears to have happened to Dapp et al. [11]. The continued output of studies using cross-lagged panel analyses suggests that knowledge of these limitations, although far from new, is lacking in the research community. Hence, continued communication of these limitations is warranted. We recommend researchers to analyze models with effects both forward and backward in time and without adjustment for the outcome variable, as we have done in the present study, in order to identify possibly spurious prospective effects.

**Table 3:** Meta-analytic cross-lagged panel analyses, and their conclusions, that we have reanalyzed and challenged.

Challenged study	Challenged claimed prospective effect	Challenging reanalysis
Gedikli et al., 2022 [21]	Reciprocal between wellbeing and unemployment (neg.)	Sorjonen and Melin, 2023 [22]
Giletta et al., 2021 [23]	Initial peer behavior on subsequent target youth behavior (pos.)	Sorjonen et al., 2023 [16]
Harris and Orth, 2020 [4]	Reciprocal between self-esteem and quality of social relations (pos.)	Sorjonen et al., 2023 [24]
Krauss and Orth, 2022 [3]	Reciprocal between self-esteem and work experiences (pos. & neg.)	Sorjonen et al., 2023 [25]
Smith et al., 2021 [26]	Initial perfectionism on subsequent depressive symptoms (pos.)	Sorjonen and Melin, 2023 [27]
Talsma et al., 2018 [28]	Initial self-efficacy on subsequent academic performance (pos.)	Sorjonen and Melin, 2023 [29]
Wang et al., 2021 [30]	Reciprocal between social support and posttraumatic stress disorder (neg.)	Sorjonen and Melin, 2023 [31]
Wu et al., 2021 [32]	Reciprocal between academic self-concept and academic achievement (pos.)	Sorjonen et al., 2022 [33]

Note: neg. = negative effect, pos. = positive effect.

## Limitations

The present study shared some of the limitations of the reanalyzed meta-analysis by Dapp et al. [11]. For example, 42 of 43 included samples were Western, from the United States, Canada, Europe, Australia, or New Zealand. A single sample was from Asia (China) and no samples were from Africa or South America. Hence, it remains an open question if the present main finding, i.e. that prospective effects between global and domain-specific self-esteem appear to be spurious due to correlations with residuals and regression to the mean, generalize to a broader cultural context.

The measurements of global and domain-specific self-esteem in the included studies may not always have been optimal. Moreover, in the present reanalyses we did not consider possible moderating influences of age and sex composition of the sample, time lag between measurements, used instruments, etc. However, it is important to bear in mind that such characteristics were constant across the analyzed models and cannot, consequently, explain why some models suggested increasing and others decreasing prospective effects between global and domain-specific self-esteem.

Extended statistical models, e.g. random-intercept cross-lagged panel models [34,35] and latent growth curve models [36,37], have been proposed as improvements of the traditional two-wave cross-lagged panel model. These extended models are presumably better at differentiating within- from between-person processes of change and, consequently, allow stronger inference about causal effects. Conflation of within- and between-person processes of change has been discussed in relation to, for example, self-esteem [38], academic self-concept [39], and self-efficacy [40]. We cannot rule out that analyses with extended and improved models would withstand scrutiny and indicate increasing effects between general and domain-specific self-esteem. Therefore, the present findings should not be seen to rule out the possible existence of such effects once and for all. Rather, and more specifically, the present study challenges conclusions by Dapp et al. [11] based on findings from meta-analytic two-wave cross-lagged panel models. It should be noted that we could not conduct analyses with extended models as they require data from more than two waves of measurement, which is what we, via Dapp et al., had at our disposal. It should also be noted that analyses of three or more waves of measurement with random-intercept cross-lagged panel models would probably not give definite answers to whether general and domain-specific self-esteem have true reciprocal prospective effects on each other or not. This because the method can be susceptible to spurious findings due to correlations with residuals and regression to the mean in a similar way as traditional two-wave cross-lagged panel models [41]. This agrees with a general conclusion that it is very difficult, maybe even impossible, to prove causal effects in correlational, i.e. non-experimental, data.

To reiterate, the objective of the present study was not to evaluate if true prospective effects between general and domain-specific self-esteem actually exist. Rather, the more limited objective was to evaluate if the data analyzed by Dapp et al. [11] allowed their conclusion that such true prospective effects exist. For this objective, our reanalyses of the data with two waves of measurement used by Dapp et al. [11], which did not allow analyses with RI-CLPM, were sufficient. Our reanalyses permitted us to conclude that the prospective effects presented by Dapp et al. [11] were spurious and, consequently, that their claims about increasing prospective effects between general and domain-specific self-esteem were not supported by their own meta-analytic data.

## Conclusions

The present reanalysis of a meta-analysis by Dapp et al. [11] found prospective effects between global and domain-specific self-esteem to be spurious, probably due to correlations with residuals and regression to the mean. Hence, reciprocal increasing effects between global and domain-specific self-esteem, claimed by Dapp et al., as well as hypotheses of top-down and bottom-up processes in self-esteem, can be called into question. It is important for researchers to be aware of the limitations of adjusted cross-lagged effects, and of analyses of correlational data in general, in order not to overinterpret findings.

## Supporting information

S1 Checklist PRISMA 2020 checklist. (PDF)
